# A case study of enhancing bidirectional feedback between research programs and an institutional community engagement advisory board

**DOI:** 10.1017/cts.2026.10780

**Published:** 2026-06-24

**Authors:** Helen H. Lee, Teresa Borowski, Marc S. Atkins, Devyani Gore, Joanna Buscemi

**Affiliations:** 1 Anesthesiology, University of Illinois Chicago, Chicago, IL, USA; 2 Institute for Health Research and Policy, University of Illinois Chicago, Chicago, IL, USA; 3 Psychiatry and Psychology, University of Illinois Chicago, Chicago, IL, USA; 4 University of Illinois Chicago, Chicago, IL, USA; 5 Psychology, DePaul University, Chicago, IL, USA

**Keywords:** community based participatory research, pilot projects, citizen science, translational science, biomedical

## Abstract

Community engagement is essential to research. Community Advisory Boards (CABs) are frequently consulted to inform recruitment and engagement strategies. In our experience, a gap emerged between CAB recommendations and implementation, largely due to limitations in research infrastructure, funding, and team capacity. Researchers may underappreciate why providing contextual details about studies that relate to resources or constraints, can lead to tailored recommendations. In response, our institutional CAB now incorporates researcher input before and after consultations to clarify programmatic and institutional limitations. This ongoing, bidirectional dialogue supports more pragmatic, tailored recommendations that better align with research team capacity while advancing shared goals.

## Introduction

Community engagement in research is rooted in relationship building. Community advisory boards (CABs) are intended to help researchers align study goals with community needs for the long-term goal of mitigating health disparities. Effective consultations require researchers to pose clearly defined questions and CAB members to provide community perspectives within the context of the research goal. Perhaps implicit is the researcher’s expectation that CAB feedback will also reflect the pragmatic limitations of the research study (e.g., budget, staffing, institutional practices). Though CAB members may not have research experience, researchers may expect CAB feedback to be readily incorporated into a complex study protocol. Experiences and expectations of CAB members have been described [1], but researcher perspective/expectations and research program infrastructure (number and profile of research team members, resources) are important factors that are less understood [2,3].

While it is increasingly recognized that consulting CABs is the “right” approach to health disparities research, the impact and value to both CAB members and research programs may be variable. Consultations with CABs provide specific recommendations to improve the relevance and engagement of biomedical research to the community. However, researchers may face barriers to implementing recommendations, particularly in early stages of building a research program. Further, while CABs and researchers may be aligned in engagement goals, creating space during consultations to discuss pragmatic implementation barriers (e.g., budget, infrastructure) may lead to more tailored recommendations. This may be particularly relevant in scenarios of new research programs with early-stage investigators and staff in the active stage of preparing for a large trial. We provide a case-based discussion of a consultation, from the perspectives and expectations of CAB and research team members, to illustrate how bilateral discussion during and after a consultation led to alterations in the structure, conduct, and preparation of future CAB consultations.

## Researcher perspective

As part of formative assessments to prepare for a pilot study of a behavioral intervention, research team members consulted our institutional Community Engagement Advisory Board (CEAB). Our agenda was to identify challenges/facilitators to participant recruitment, engagement, and retention. To prepare the CEAB, we provided scientific background and context for the research and the pilot study. We did not provide any context related to logistics and operations, in part because we did not consider how this would influence CEAB recommendation and in part because we did not anticipate many of the operational barriers. In hindsight, pre-consultation probes to consider the pragmatic aspects of conducting a pilot would have benefited both researchers and CEAB members during the consultation. We present some of the operational issues to help readers anticipate how our team could have better prepared the CEAB for consultation. The research team had one year to conduct an 8–10-month pilot study. Funding for a five-year clinical trial was contingent upon demonstrating research milestones, as set in agreement with the funding agency. Other pertinent context was that the clinical space of recruitment (dental clinic) was not the clinical home (hospital-based anesthesiology) of the principal investigator (PI). Because research activities involved patients and clinical environments outside the PI’s clinical department, preparing for research activities also included stakeholder meetings with research leaders, clinical faculty, trainees, clinical staff, administrative staff, information technology, and marketing and human resources. Concurrently, the contact PI was actively building the research program’s infrastructure, which included operational processes such as establishing payment accounts to conduct research and operations within an academic medical center and state institution. Navigating institutional processes to hire staff or execute purchases with institutional-approved vendors was more labor intensive than anticipated. Due to budgetary constraints we planned to utilize volunteer research assistants, primarily undergraduate college students, to carry out recruitment and enrollment activities. The remaining gap in the study protocol lay in the recruitment strategy. While members of our research team have conducted short term studies with the same population [4,5], there was no institutional experience recruiting the study population for an 8–10-month pilot study.

## Community advisory board perspective

A CEAB is different from a CAB in that it contains community expertise across clinical and research domains whereas a CAB would be formed to advise a specific research project or program [6]. An institutional CEAB differs from a traditional project specific community advisory board in that it advises on a variety of research projects and consists of diverse membership in expertise, demographics, and roles [7]. Further, unlike a project specific community advisory board, a CEAB can support capacity building for community-engaged research among academic and community partners, for example, by enhancing bi-directional relationships between community members and academic researchers. Additionally, the increase of active engagement of community experts in health research CABs has shown the potential to amplify impact and have reshaped scientific expectations, evolving into the role of advisory boards as more long-running organizations with broader scope and value [7].

An additional goal is to provide investigators with guidelines on developing their own CAB if needed. CEAB members represent lay community members, leaders of community organizations, research staff, and researchers and participate in ongoing training on basic understanding of research processes and terminology, and core principles of community-based research [8]. A description of the formation and implementation of the CEAB can be found at Matthews et al. 2018 [9].

In preparation for a consultation, CEAB members are usually provided a lay language summary of the research project, current recruitment strategies and recruitment materials, if relevant. Additionally, they are informed of the nature of feedback requested from the research team, e.g., recruitment/engagement strategies. Important background and context for researchers to provide to CEAB members include funding mechanisms, budget, timeline of project. However, this context is often provided at discretion of individual researchers and often with minimal appreciation of CEAB member background, training, experience, or perspective. CEAB members were encouraged to provide candid feedback on existing recruitment/engagement strategies as well as proposing their own suggestions based on socio-economic, cultural, racial and ethnic identities of communities in Chicago.

## Reflections on incorporating CEAB feedback into pilot study

Recommendations: A few weeks after the 60-minute consult, a summary of recommendations was sent to the researchers via email. Suggestions to improve participant engagement fell into the following categories: access, awareness, racism, workforce diversity. Within categories, CEAB provided recruitment strategies that would enhance participant engagement by mitigating challenges. Recommendations to improve participant engagement and the PI’s perspectives on challenges to implementation and the response or pilot study experience is summarized in Table 1.

**Table 1.** Overview of CEAB recommendations, challenges to uptake, and research program experience, by domains of participant engagement Table 1 long description.

Participant engagement	CEAB recommendation	Challenges	Pilot study response/experience
Access	Transportation Recruit in person at the clinic Recruitment activities at Back-to-School events, health fairs, places of worship, newspaper advertisements	Lack of funding to staff clinic recruitment Study population narrowly focused based on clinical criteria	Student volunteers recruiting in clinic Recruitment efforts focused at clinic not community level
	Caregiver burden Pre-screen families for anticipated retention issues during study period Ask participants best mode of communication, Access to phone services may be unreliable due to cost Tangible benefits to children, e.g., rewards such as an electric toothbrush	Purpose of pilot study was to explore retention issues that affect feasibility and acceptability of clinical trial Asking for preferred mode of communication was insufficient to enhance recruitment No budget to provide children tangible benefits	PI donated toys for children
	Opportunity cost Compensate families after intervention visit.	Compensation after intervention vs. data collection could introduce financial incentives to adhere to intervention and interfere with measuring impact	Recruitment materials included specific compensation amounts and types of visits (data collection vs. intervention)
Awareness	Contact with healthcare system Transportation vouchers for all dentist appointments during study period Social media to share study information Introduce the study via mail/email/electronic health record	Request to purchase institutional parking ticket vouchers delayed by internal administrative processes. Study population narrowly defined by presenting to clinic	Parking ticket vouchers lagged pilot study recruitment by 3 months after majority participants already enrolled Study participants accessed the clinic’s website to find care. A short article and video about the pilot study was integrated in the clinic’s website
	Language & health literacy Professional translation of all study documents	Budget	chat GPT utilized for Mexican Spanish translations at 5^th^ grade literacy level, reverse translated by bilingual research assistant
	Research literacy More details about compensation, risks/benefits, and time commitment Hire a community outreach specialist	No challenges Budget	Compensation schedule included in recruitment/enrollment documents
Racism	Interpersonal Participant confidentiality/eligibility for public programs Mistrust that poor oral health=negligence; historical association of social workers with police/reporting Informational fliers & posters include representative images of study population	Issues of trust in research or health-related services may be deeply rooted in communities	Informed consent contains explicit language=participation status not related to eligibility or participation in other public programs. Research program and social work overlap discussed with the dental clinic social worker and clinical psychologist. CHW weekly meetings to discuss participant psychosocial issues. Recruitment materials contain images representative of clinical population
	Structural Poor Medicaid coverage of dental care	How to disseminate research activities and findings to policymakers? Prioritization of resources toward research milestones vs. addressing Medicaid policies	
Workforce diversity	Lack of recruiter diversity CHW vs research assistant recruiting CHWs obtain consent	Budget Hiring CHW exceeded 6 months due to institutional and individual delays	Volunteer RA’s, underrepresented in science, trained to recruit and enroll

The CEAB recommendations emphasized facilitating participant access by providing transportation to activities (e.g., public transit fare cards); reducing burden on caregiver time; and consideration of the opportunity cost of participating in intervention visits (e.g., release from work, child care needs). One example was to embed recruitment staff in public spaces easily accessible to participants (e.g., home-based, or community sites such as parks or schools). However, while these suggestions may be appropriate for many community-based studies, our pilot study population was specific to families that presented for care in the dental clinic.

Similarly, acknowledging caregiver burdens that related to daily activities or stress by asking participants for preferred mode of communication (phone vs. video conference) was difficult to implement given that, in this study, most participants mode of communication tended to fluctuate throughout the study. Additionally, preferred mode of communication also depended on purpose. For example, to arrange a CHW session, we found that while all participants preferred communicating via text message. However some participants, especially those with families in other countries, preferred to text using applications such as WhatsApp, which wasn’t specifically probed by staff. However, the ability to identify families with possible retention issues overlapped directly with the characteristics of the study population (Medicaid enrolled families in urban Chicago neighborhoods who may be experiencing housing, financial, and food security). Opportunity costs of participating in research are high for our pilot study population, which was the rationale for CEAB recommendation to compensate for intervention visits. However, the scientific team recognized the possible introduction of financial incentives that could bias participant adherence to intervention visits and also introduce inequities between intervention vs. usual care arm participants.

Participant awareness of research opportunities and understanding of risks/benefits of participation were addressed through CEAB recommendations included introducing research within the context of interaction with the healthcare system; addressing language and health literacy levels to align with the population; and overcoming possible research literacy issues. While providing parking tickets for dental visits appeared to be a straightforward task, the administrative processes of purchasing was so delayed that the majority of pilot study participants had enrolled by the time vouchers were available. To address language and health literacy, the use of professional translation services for all study documents was prohibitive within our budget. Our modified approach was to utilize chat GPT to translate from English to Spanish. A bilingual volunteer research assistant reverse translated all documents from Spanish to English.

Racism, experienced at the interpersonal and structural levels, was identified as barriers to research engagement. Our study population’s lived experiences include navigating health care through the lens of structural and interpersonal racism. CEAB suggestions to acknowledge racism ranged from granular (representative images in informational fliers) to policy-level (discussion around influencing policy to increase access to care). While our team acknowledged these barriers, it was a challenge to identify and implement strategies to mitigate the impact on research engagement beyond reflecting the population within recruitment materials and study documents. We acknowledge the gaps in Medicaid coverage and access to dental providers, which influenced recommendations to apply scientific findings to advocacy and policy.

Finally, CEAB recommendations identified the importance of diversity in the recruiter workforce. The recommendation to assign a community health worker, who is the study interventionist, to also serve a role in recruitment and enrollment activities was recommended as an important strategy to improve participant engagement. While this is a strategy we are currently employing in an ongoing clinical trial, budget and hiring issues precluded us from implementing this during the pilot study. However, we did ensure that volunteer research assistants were representative of the study population.

Research team leaders (HL, JB, TB) selected feedback items to incorporate into the protocol based on feasibility of budget, staffing, and resources. Barriers to implementation were identified and communicated to CEAB in a follow up survey. This instigated changes in the process of CEAB consultations, namely asking researchers to identify funding source, phase of study, and CEAB member training to understand implications of this information on research programs.

## Discussion

Community engagement is a critical factor to recruitment for clinical trials, mitigating health disparities [10,11], and the translation of research into practice [12]. Collaboration between CEABs and research programs relies on bi-directional feedback. While CEABs are designed to provide community perspectives to inform research, reciprocating feedback from research teams to the CEABs is often a missed opportunity to refine consultations. The impact of these missed opportunities could lead to misperceptions about the relevance or feasibility of implementing CEAB feedback.

Soliciting feedback from a CEAB garners valuable strategies, which might be implemented in stages over time. As a result of this consultation session, the Community Engagement and Collaboration Core implemented the following changes to further equip CEAB members with the context and knowledge they may need to provide constructive feedback to research teams. In preparation for each consultation session, CEAB members now receive the specific source of funding that the research team has or will be applying to and are provided with specific information about study phase. This information is reiterated during the start of the consultation session. A resource directory has been developed for all CEAB members with lay language articles and resources about research terminologies, protocols, research design. Additional training sessions have been implemented to keep CEAB members up to date on key aspects of research processes, such as (1) Overview and characteristics of funding mechanisms in health research (NIH grants vs non-NIH); (2) Academic vs community based methods of dissemination of study findings; (3) Overview of implementation science research and the role of community perspectives in informing the generation of evidence-based practices; and (4) Overview of IRB submissions by research teams. Finally, in addition to the above topics and processes, we recommend modifying CEAB consults by including following information for consideration: sources of constrained staffing/resources and research program short term vs long term goals, to help guide scope of recommendations.

We highlight our experiences engaging with a CEAB. At the time of writing, we have completed our pilot study and have the luxury of implementing many of the CEAB recommendations in the absence of budgetary and staffing constraints. At the point of the pilot study, implementing some CEAB recommendations were not feasible because they were beyond the pilot study budget or staffing. However, years later, CEAB suggestions informed the protocol for a clinical trial which is associated with a larger budget, greater staffing and other resources (e.g., defined collaborative relationships with clinical partners). With additional time, we were able to coordinate recruiting and enrolling within the clinical delivery system, including informational flyers in patient folders and asking clinical schedulers to tell patients about a potential recruitment phone call. With a larger paid research team, we are able to utilize community health workers (interventionists) to assist with recruitment and enrollment. Finally, the experiences of our pilot study have given us the perspective to reflect upon our roles as advocates. CEAB members identified inadequate access to dental care, disproportionately affecting under-resourced communities, as a structural issue for the research team to address. Initially, this appeared outside the scope of a pilot study. Over time, this suggestion has become a call to action. Research leadership (HL, JB) now frame our community engaged research to include policy advocacy. CEAB members indicated, through their feedback, that the knowledge gained through research carries a responsibility to advance knowledge in a meaningful way for the communities we study and serve.

A university-wide CEAB, provides an efficient mechanism to improve recruitment and retention strategies, especially during a time of funding restrictions. However, as a university-wide program, the feedback may not be sensitive to elements of a program of research such as unique setting and population features. Creating opportunities for follow-up input to CEABs could improve the capacity of CEAB members to provide pragmatic guidance and feedback to researchers. Feedback could reflect tiers of participant engagement/recruitment/retention activities, allowing researchers with a range of options when budgets and other resources are dynamic.

Our experiences, while described in the specific context of one institution, have a high degree of generalizability. Biomedical research is funded by a wide range of public and private sources. However, the need for community engagement in biomedical research remains a consistent priority. As the availability and capacity of public funding grows constrained, the ability of community members to engage and influence how, where, and why biomedical research is conducted will increasingly rely on productive dialogue between CABs and researchers. Our experiences highlight the benefits of continued engagement between researchers and CABs throughout the consultation process.

## Table 1. Long description

The table presents a framework for adapting community-based participatory research recommendations to a pilot study, categorized by barriers to research participation (access, awareness, racism, and workforce diversity). Across all domains, the most common challenges to adapting the recommendations related to limited funding, staffing constraints, recruitment difficulties, and balancing community priorities within a limited research infrastructure. Proposed adaptations focus on flexibility, community engagement, incorporation of community health workers, multilingual communication, and ongoing evaluation to determine which approaches improve access, participation, and outcomes.

Navigate back to Table 1..
